# Point of Care Ultrasonography for the Septic Patient in the Emergency Department: A Literature Review

**DOI:** 10.3390/jcm12031105

**Published:** 2023-01-31

**Authors:** Christos Verras, Ioannis Ventoulis, Sofia Bezati, Dionysis Matsiras, John Parissis, Effie Polyzogopoulou

**Affiliations:** 1 Emergency Department, National and Kapodistrian University of Athens, Attikon University Hospital, 12462 Athens, Greece; 2 Department of Occupational Therapy, University of Western Macedonia, 50100 Kozani, Greece

**Keywords:** emergency department, point-of-care ultrasound, sepsis, septic shock

## Abstract

The point-of-care ultrasound (POCUS) has been effectively used in intensive care units for the management of septic patients. Since it is a time- and cost-effective non-invasive imaging modality, its use in the emergency department (ED) has been advocated for by medical experts. This review summarizes the existing literature regarding the breadth of POCUS as a supplementary tool to the holistic approach of septic patients in the ED setting. A literature search was conducted via PubMed (MEDLINE), Cochrane Library, and Scopus databases, analyzing studies which examined the use of POCUS in the ED for non-traumatic, septic, and/or undifferentiated hypotensive patients, resulting in 26 studies. The first cluster of studies investigates the efficiency of POCUS protocols in the differential diagnosis and its reliability for distributive/septic shock and sepsis management. In the second cluster, POCUS use results in faster sepsis cause identification and improves therapeutic management. The third cluster confirms that POCUS aids in the accurate diagnosis and management, even in rare and complicated cases. The results of the present review support the well-documented utility of POCUS and highlight the importance of POCUS incorporation in the comprehensive management of the septic patient in the ED setting.

## 1. Introduction

Sepsis is a medical emergency and a life-threatening disorder, which imposes a major health burden on emergency departments (EDs) and intensive care units (ICUs), accounting for a significant proportion of in-hospital and ICU mortality [1]. In a recent analysis of the Global Burden of Disease, Injuries, and Risk Factors (GBD), sepsis morbidity and mortality is estimated to 48.9 million global cases and 11 million deaths, or 1 in 5 deaths worldwide. Of the 48.9 million cases, 33.1 have occurred in patients with an underlying infectious condition and the remaining 15.8 million in individuals with underlying injuries or non-communicable diseases (NCD) [2]. Although the initial interest on sepsis has been focused in the ICU setting, attention has been gradually shifted towards the ED field, since it has been recognized that the early initiation of treatment in the ED [3], ideally within one hour from the diagnosis of sepsis, is crucial in order to decrease length of stay, morbidity, and mortality [4].

To this end, point-of-care ultrasonography (POCUS) has emerged as an adjunct imaging modality for the management of the septic patient upon arrival to the ED. POCUS allows for fast, real-time assessment of cardiovascular, respiratory, or other acute pathologies. Moreover, as an extension of physical examination and in conjunction with additional testing, it narrows differential diagnosis. In this way, it enhances the initial management plan and it shortens the time to clinical decision-making. Apart from its diagnostic value, POCUS can also provide information regarding a patient’s response to treatment when serially performed [5,6]. Ιn the 1990s, emergency medicine (EM) physicians in the United States of America advocated for the use of POCUS in the ED, so POCUS nowadays is a standard component of EM residency training programs [5]. In light of the recognition of the usefulness of POCUS in EM, several ultrasound protocols have been developed and implemented. The most well-known and widely used protocol for non-traumatic patients is the rapid ultrasound in shock (RUSH) exam, which helps clinicians differentiate various etiologies of shock in a short period of time [6,7,8]. 

Considering the enormous sepsis-induced burden on health care systems [2], in conjunction with the complex nature of sepsis-associated causes and symptoms, it is obvious that the integration of methods and techniques for early diagnosis and management of sepsis in the ED is not just useful, but also of critical importance. In view of the aforementioned facts, the aim of this manuscript is to review the existing literature regarding the breadth of POCUS use and its contribution to the prompt diagnosis and effective management of septic patients in the ED setting. Specifically, the present review focuses on the existing data with reference to the utility and reliability of multimodal POCUS for non-invasive differential diagnosis of shock, rapid identification of the septic source, and treatment guidance.

## 2. Materials and Methods

A literature review was conducted using the online databases PubMed (MEDLINE), Cochrane Library, and Scopus. In our search strategy, the publication period under consideration was defined from 2010 to July 2022, excluding all grey literature. 

The combinations of medical subject headings that were used in searching included the following: [Sepsis OR Septic Shock OR Septic OR Undifferentiated Shock OR Hypotensive Patient] and [Emergency Department OR ED OR Emergency Room OR Emergency Medicine] or [Point-of-Care OR Bedside OR POCUS] and [Ultrasonography OR US]. In addition to the database search, the cited bibliography of the selected articles was reviewed in order to ensure that no significant relevant research data were missed. 

Three reviewers worked independently to review all eligible titles and abstracts, and by using inclusion criteria, defined a priori. These criteria were: publication language (English), time of publication (2010–July 2022), aim (assessment of POCUS use in the ED for non-traumatic, septic, and/or undifferentiated hypotensive patients), and research type, namely randomized clinical trials (RCTs), observational studies (including both prospective and retrospective cohorts), and case reports. After initial selection, the reviewers read the full text to determine whether inclusion criteria were met.

## 3. Results

**Figure 1:**
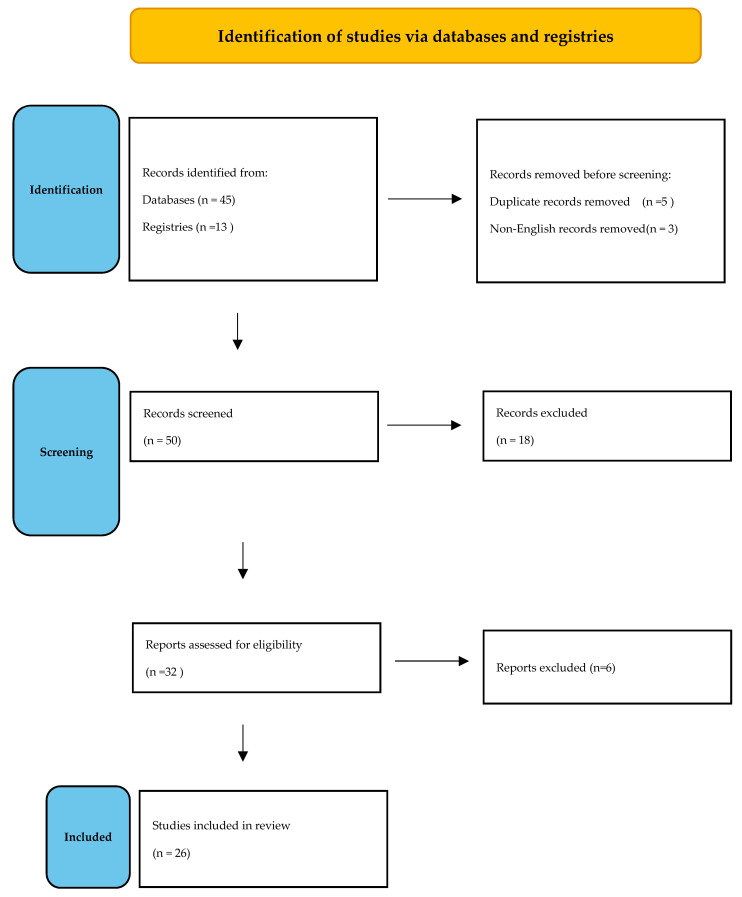
Search flow diagram.

Initial search resulted in a total of 58 articles, with 32 being eligible for review. After reading the full text, 6 more articles not meeting inclusion criteria were excluded. Eventually, 26 articles [9,10,11,12,13,14,15,16,17,18,19,20,21,22,23,24,25,26,27,28,29,30,31,32,33,34] were included in the final analysis (Figure 1). 

Out of 26 articles, 11 were prospective observational studies, 2 were RCTs, 1 was a retrospective cohort trial, and 12 were case reports. All but two ([10,14]) were single-center studies. Except for the case reports, the remaining 13 articles were divided into two categories based on their main purpose: the first cluster comprised 9 articles [9,10,11,12,13,14,15,16,17] which studied the contribution of POCUS to the diagnosis of undifferentiated hypotensive/critically ill, non-traumatic patients (Table 1); the second cluster consisted of 5 articles [18,19,20,21,22] which studied the utility of POCUS in determining sepsis cause and managing those patients (Table 2). The main results of the 12 case reports [23,24,25,26,27,28,29,30,31,32,33,34] are presented in Table 3.

## 4. Discussion

Although sepsis represents one of the main causes of ED admission, its incidence remains underestimated because it constitutes an intermediate and not a primary cause of illness and death [2]. In the present review, the studies included in the first cluster demonstrate that the incidence of sepsis remains high, yet widely variable. Specifically, three [9,11,12] out of nine studies present the frequency of septic shock in the ED (3.6%, 12%, and 13.5%, respectively), while four [10,13,15,17] report the overall incidence of in-hospital sepsis (52%, 32%, 22.2%, and 55.5%, respectively). All these studies emphasize the importance of recognizing sepsis early in the clinical course, while consistently adopting a systematic evidence-based bundle of care and implementing it in a timely manner. Indeed, this approach is corroborated by recent sepsis guidelines [4]. 

Almost all studies from the first cluster conclude that the use of POCUS contributes to the early differential diagnosis, either of the cause of hypotension, or the cause of signs and symptoms that are suggestive of shock regardless of systolic blood pressure. This notion is even supported by Atkinson et al. [10], although their RCT failed to demonstrate any survival benefit conveyed by the use of POCUS in ED patients with shock and undifferentiated hypotension. In addition, in four out of nine studies, the addition of POCUS to standard ED care yielded a narrower and more accurate list of possible causes of nontraumatic undifferentiated hypotension, as evidenced by the reliability indices displaying agreement between the initial working diagnosis (based on combined clinical and POCUS evaluation) and the final diagnosis. Indeed, with regard to distributive shock, the combined clinical and POCUS diagnosis demonstrates high agreement with the final diagnosis (as measured by Kappa coefficient—k, ranging from 0.71 to 1.00), while it shows very good sensitivity (63.6–75%), as well as excellent specificity (99.7–100%), positive predictive value (87.5–100%), and negative predictive value (86.1–100%) [9,11,12,13] (Table 4). Two key points of these studies are worth mentioning. First, when implementing RUSH protocol, serial examinations are needed in cases of suspected septic shock [12]. Second, sepsis is a form of distributive shock that can be promptly diagnosed through identification of the sepsis source with the use of POCUS; multimodal POCUS can reveal multiple “foci of sepsis”, such as consolidation, air bronchogram, gallbladder wall thickening, limb cellulitis, hypoechoic pancreas, and vegetations [13]. Furthermore, Sasmaz et al. [15] and Volpicelli et al. [17] have reported perfect total agreement between the preliminary (ultrasonography-based) and final diagnosis (k = 0.82 and k = 0.838 respectively, *p* < 0.001 in both). In the latter study, ultrasonography-based diagnosis for distributive shock was accurate in 35 of 40 patients (87.5%). In conclusion, the contribution of POCUS to the early diagnosis of nontraumatic undifferentiated hypotension in ED patients is almost indisputable. Even in the sole study with contradictory results, which reports that POCUS use in the ED prior to a key intervention was associated with a higher mortality rate in critically ill patients [14], the authors admit potential sources of bias against POCUS. In fact, their registry does not include patients who were resuscitated in the ED. On top of that, the proportion of patients with diagnostic uncertainty, who were eventually identified as critically ill with the use of POCUS, is not taken into account. 

The second cluster of studies [18,19,20,21,22] acknowledge the contributing role of POCUS in determining the source and guiding the management of sepsis. Considering the complex nature of sepsis, the results are really promising. According to a study by Cortellaro et al., in 89% of the cases, POCUS enhanced emergency physicians’ (EP) ability to identify the cause of sepsis; moreover, POCUS-based diagnosis was achieved in an expedited manner (10min), with great accuracy (75%) and sensitivity (73%) [18]. Furthermore, the use of POCUS has been shown to be beneficial in terms of guiding fluid administration and achieving hemodynamic improvement in 97.4% of ED patients with septic shock [19]. In the study of Haydar et al., it was demonstrated that the use of POCUS facilitated clinical decision-making by increasing EPs’ certainty in 71% of the cases and by leading to the revision of overall treatment plans in 53% of the cases (change of the presumed cause in 17% and modification of the procedural intervention plans in 27%) [20]. However, a recent RCT assessed the effect of ultrasound-guided fluid management (UGFM) in patients with septic shock and showed no significant difference in 30-day mortality between patients receiving usual care and patients of the UGFM group, even though the latter group were administered less amount of resuscitation fluid [21]. Finally, the fourth study of this cluster [22] pointed out the high prevalence of sepsis in ED patients with acute circulatory failure (ACF) and verifies the preponderance of hypovolemia and vasoplegia in septic patients. This study showed that focused cardiac ultrasounds (FOCUS) could promptly identify left or right ventricular systolic failure in 31% of septic patients. Additionally, the study depicted that, even recently trained in ultrasounds, EPs were able to properly identify ACF mechanisms, achieving high agreement with the interpretation of ultrasound findings by ICU experts. Therefore, early FOCUS assessment can serve as a guide to fluid resuscitation in septic patients presenting to the ED with ACF.

Similar promising results have emerged from the third cluster of studies, which included case reports. POCUS was found to be a supportive tool for early and accurate diagnosis and overall management in complex septic cases. Three of the POCUS-based diagnoses involved the cardiovascular system (acute heart failure with reduced ejection fraction, severe aortic stenosis, and mitral regurgitation [23]; myocardial infarction caused by endocarditis-related septic embolization [26]; methamphetamine-associated cardiomyopathy [30]). In the latter case, POCUS set the diagnosis, dictated the appropriate restriction in the administration of intravenous fluids, and guided proper consultation. Two cases involved the respiratory system. Τhe first one was a ruptured pulmonary hydatid cyst, in which case POCUS directed EPs towards a timely and proper diagnosis, hence enabling appropriate management of the underlying septic and anaphylactic shock while prompting definitive surgical intervention [29]. The second one was an unexpected empyema revealed by POCUS in a patient being treated for community-acquired pneumonia [33]. Both of these cases confirm the utility of POCUS, not only in diagnosing pneumonia, but also in identifying associated complications, such as pleural effusions or empyema. Accordingly, POCUS is proposed as a valuable tool in the ED setting for patients with undifferentiated dyspnea and sepsis. Four cases dealt with various other clinical entities, namely esophageal perforation [27], obstructive uropathy causing moderate kidney hydronephrosis [25], xanthogranulomatous pyelonephritis [28], and liver abscess [34]. Regarding esophageal perforation, deterioration and death due to sepsis can occur within hours of presentation. Likewise, obstructive pyonephrosis has a mortality rate of 40% if not addressed in a timely manner, while pyogenic liver abscess is also accompanied by high mortality if not diagnosed promptly. In all previous four cases, POCUS imaging played a pivotal role in the accurate diagnosis of the sepsis cause. Finally, two case reports and a retrospective case series analysis involved the musculoskeletal system: necrotizing fasciitis of the left inner thigh, diagnosed via RUSH protocol [24]; glenohumeral joint septic arthritis and subdeltoid septic bursitis, in which case musculoskeletal POCUS was diagnostic through ultrasound-guided needle aspiration [32]; and septic arthritis of the hip diagnosed via POCUS and treated with aspiration in the ED in a series of 17 children [31]. 

All the aforementioned studies emphasize the utility of POCUS in the ED, highlighting its role as a time- and cost-effective, accurate, and non-invasive diagnostic imaging modality. With the focus on septic patients and their complex pathology, a systematic effort is warranted in order to develop integrated protocols [8,9], algorithms [25], and guidelines. The objective is to facilitate the rapid and holistic assessment οf patients in the ED, readily identify the source of sepsis or the cause of undifferentiated hypotension, and further guide the resuscitation process. A recent review summarizes the application of an integrated bedside ultrasound multiorgan approach in septic patients as an adjunctive tool to clinical examination and laboratory studies. Its use is not only supported for the diagnosis of septic shock and for the identification of the culprit infection, but also for the assessment of fluid responsiveness, resuscitation effectiveness, and guidance of diagnostic procedures and infectious source control [35].

This review depicts the limited number of studies regarding the use of POCUS in ED septic patients when compared with similar studies in the ICU setting, which is the main limitation of the study. These available studies have shown diversity in terms of cohort size and characteristics, POCUS parameters evaluated, and outcome elements that have been measured. However, even though POCUS is an established imaging modality in the ED for multiple time-sensitive, critical diseases such as trauma or cardiac arrest and it is already incorporated in universal guidelines, it seems that there is a delay in the integration of POCUS in international sepsis guidelines.

## 5. Conclusions

The results of the present review support the well-documented utility of POCUS and highlight the importance of POCUS incorporation in the comprehensive management of the septic patient in the ED setting. At the same time, literature remains limited in terms of large scale, multi-center studies focused only in septic patients presented to the ED. Further development of research studies, alongside with integration of POCUS use in sepsis guidelines, will strengthen and extend its use in everyday clinical practice.

## Figures and Tables

**Table 1:** Studies examining the contribution of POCUS to the diagnosis of undifferentiated hypotensive/critically ill patients *.

1st Author, Year, Country, Design, Setting	POCUS Protocol (If Any)	Aim	Patient Number/Age	Main Results
Ahn et al., 2017, Korea, POS, single- center ED [9]	SEARCH 8E’s	“SEARCH 8E’s” protocol vs. final diagnosis	308/>18 yo	For sepsis: Sensitivity 63.6%, Specificity 99.7%, PPV 87.5%, NPV 98.7%, k = 0.729 (*p* < 0.001)
Atkinson et al., 2018, international (N. America & S. Africa), RCT, multicenter (n = 6) [10]	Μulti-organ POCUS based on ACES & RUSH protocols	POCUS protocol vs. standard care without POCUS	273/>19 yo	No benefit in survival, LOS, rates of CT scanning, inotrope use, or fluid administration The diagnosis in >50% was occult sepsis
Bagheri-Hariri et al., 2015, Iran, POS pilot, single-center ED [11]	RUSH	RUSH-based shock type diagnosis vs. final diagnosis	25/N/A	Agreement rate between RUSH-based and final diagnosis for the indexes APACHE II, CVP and LVEF, was 100% for all shock types
Ghane et al., 2015, Iran, POS, single- center ED [12]	RUSH	Accuracy of early RUSH protocol performed by emergency physicians to predict shock type in critically ill patients	52/>18 yo	Overall k = 0.7 (*p* < 0.001) Distributive shock (87.5% with sepsis): k = 0.83 (*p* < 0.001), Sensitivity = 75%, Specificity & PPV = 100%, NPV = 94.9%
Javali et al., 2020, India, POS, single—center ED, 18-month period [13]	Multi-organ POCUS protocol	Multi-organ POCUS to improve accuracy, narrow differential diagnosis, test effectiveness of EGDT	100/>18 yo	Accuracy of Clinical vs. POCUS diagnosis was 45% and 47% respectively, for combined Clinical &POCUS 89% Clinical &POCUS: agreement with final diagnosis was k = 1 for obstructive shock patients, k = 0.717 for distributive shock and k = 0.89 for all included patients Distributive shock: 38% (84% with sepsis) Reliability indices of C&P: Sensitivity 73.68%, Specificity & PPV 100% and NPV 86.11%. POCUS was accurate for sepsis (foci of sepsis identified) Hyperkinetic LV in POCUS is an independent predictor of septic shock
Mosier et al., 2019, USA, ROS (cohort), 2-center EDs [14]		Impact of POCUS on care processes and outcomes in critically ill nontraumatic patients Method: 3 patient cohorts: no POCUS (cohort 1 = 4165), POCUS prior to key intervention (cohort 2 = 614), and POCUS after key intervention (cohort 3 = 662). Primary outcome: in-hospital mortality	5441/> 18 yo	Mortality for cohorts 1, 2, 3: 22%, 29%, and 26% respectively (*p* < 0.001) Cohort 2: adjusted OR for death = 1.41 (95% CI, 1.12–1.76) compared to Cohort 1 Septic patients’ mortality: 29.2%, 43.9% &27.1% for cohorts 1,2,3 respectively (*p* = 0.002)
Sasmaz et al., 2017, Turkey, POS, single-center ED [15]	RUSH	Effect of POCUS on clinical decision, by comparing diagnosis before and after POCUS with the definitive diagnosis	180/>18 yo	Consistency for preliminary and post-POCUS diagnosis: 60,6% [k = 0.564 (*p* < 0.001)] & 85% [k = 0.82 (*p* < 0.001)] respectively Post-POCUS: Treatment plan modification in 50% of patients Change of preliminary diagnosis in 27.9% of septic patients
Shokoohi et al., 2015, USA, POS, single-center ED, 32-month period [16]	US hypotensionprotocol (FOCUS, RV, IVC, abdominal & transthoracic scans)	Impact of protocol on diagnostic certainty & ability, treatment, and resource utilization	118/>18 yo	Diagnostic uncertainty: −27.7% (In sepsis alone: −21.1%)Absolute proportion of definitive diagnosis: +11.9% Changes: treatment plan 24.6%, further diagnostic imaging 30.5%, consultation plan 13.6%, admission level of care 11.9% Concordance with the blinded consensus final diagnosis k = 0.80
Volpicelli et al., 2013, Italy, POS, single-center ED [17]	Multi-organ POCUS protocol	Efficacy of protocol, for diagnostic process of symptomatic, hypotensive patients in the ED Assessment of decisive role of included lung scan	108/N/A	Overall concordance between POCUS-based and final diagnosis: k = 0.710 (and after eliminating the indefinite final diagnoses k = 0.971) Lung examination was decisive for the definite diagnosis of more than 20% of the cases The study revealed characteristic US patterns for distributive and hypovolemic/distributive shock Sepsis accounted for 55.3% of the cases

* All studies excluded traumatic patients and/or hypotension due to obvious cause. Abbreviations: ACES = Abdominal and cardiac evaluation with sonography in shock; APACHE II = Acute physiology and chronic health evaluation II; CI = Confidence interval; CT = Computerized tomography; ED = Emergency department; CVP = Central venous pressure; EGDT = Early goal-directed therapy; FOCUS = Focused echocardiography; IVC = Inferior vena cava; k = Kappa coefficient; LOS = Length of stay; LV = Left ventricle; LVEF = Left ventricular ejection fraction; N/A = Not available; NPV = Negative predictive value; OR = Odds ratio; POCUS = Point-of-care ultrasound; POS = Prospective observational study; PPV = Positive predictive value; RCT = Randomized controlled trial; ROS = Retrospective observational study; RUSH = rapid ultrasound in shock; RV = Right ventricle; US = Ultrasound; SEARCH 8E’s = Sonographic evaluation of etiology of respiratory difficulty, chest pain and hypotension using “8 Es’”: empty thorax, edematous lung, extended focused assessment with sonography for trauma (E-FAST), effusion, equality (left to right ventricular ejection fraction ratio), exit (aorta), entrance (IVC) and endocardial movement [8]; vs. = Versus; yo = Years old.

**Table 2:** Studies examining the utility of POCUS in identifying the source of sepsis, as well as guiding and managing septic patients *.

1st Author, Year, Country, Design, Setting	POCUS Protocol (If Any)	Aim	Patient Number/Age and Main Inclusion Criteria	Main Results
Cortellaro et al., 2017, Italy, POS, single-center ED [18]		Comparison of standard diagnostic work-up vs. early POCUS use regarding speed of diagnosis and accuracy in identification of the infectious source	200/>18 yo	Post-POCUS identification of sepsis cause: sensitivity 73%, accuracy 75%. All post-POCUS diagnoses obtained within 10 min Source-related sensitivity post-POCUS: pneumonia > 90%, soft tissue infection and cholecystitis ≈ 80%, diverticulitis and appendicitis ≈ 60% Change of antimicrobial therapy post-POCUS: 24% Overall identification of sepsis source: 89%
Devia Jaramillo et al., 2021, Colombia, POS cohort, single-center ED [19]	USER	US-based protocol for fluid administration and initiation of vasopressors in septic shock.	83/>18 yo in septic shock	Statistically significant difference in fluid balance: ○ at 4 h: standard care median 1325 mL vs. USER use 900 mL ○ at 6 h: standard care median 1658 mL vs. USER use 1107 mL ○ total fluid balance of hospital stay: standard care median 14,564 mL vs. USER use 8660 mL With USER, MAP ≥ 65 mmHg was achieved in 97.4% within 4 h
Haydar et al., 2012, USA, POS, single-center ED [20]	Protocol consisting of 3 main POCUS measures	Effect of 3 POCUS measures on clinical decision-making	74/>18 yo	Sepsis 37%, severe sepsis 40%, Septic Shock 22%, SIRS 1% Post-POCUS change of Certainty, measured in 100 mm VAS for sepsis, severe sepsis, septic shock, SIRS respectively: ○ about the cause: +5.6, 12.7, 9.5, 4 mm ○ about planned interventions: +0.3, 9.9, 2.2, 4 mm ○ about interventions foreseen: +12.3, 1.5,6.4, 7 mm ○ about choosing correct series of interventions: +9.9, 5.1, 7.8, 8 ○ about disposition: +8.1, 7.8, 5.4, 7 mm Overall Certainty change: (+): 71%, (−): 29% Change of the cause: 17% Change of the procedural intervention plans: 27% Change of overall treatment plans: 53% Mean clinical utility score: 65 mm, with usefulness reported in all cases
Musikatavorn et al., 2020, RCT, single-center ED [21]	IVC assessment	Effect of UGFM strategy on 30-d mortality in patients with septic shock or sepsis-indued hypoperfusion vs. standard care.	202/>18 yo	no significant difference in 30-day overall mortality s (18.8% and 19.8% in the usual-care and UGFM strategy, respectively; *p* > 0.05) less volume of cumulative fluid administered in the UGFM compared to standard care study group (1.900 mL vs. 2.600 mL the first 6 h, respectively, *p* < 0.001)
Lafon et al., 2020, France, POS, single-center ED [22]	FOCUS	FOCUS-based evaluation of early hemodynamic profile in patients presenting with ACF	100/>18 yo presenting with ACF	Sepsis cohort: 55 patients, Non-Sepsis: 45 patients. FOCUS was performed after administration of 500 mL of crystalloids Patients with sepsis had qSOFA score ≥ 2 points on ED admission and: More frequent CNS dysfunction Significantly increased heart rate and hemoglobin level LV hyperkinesia associated with profound vasoplegia and hypovolemia Reduced IVC size

* All studies included non-traumatic septic patients. Abbreviations: ACF = Acute circulatory failure; CNS = Central nervous system; ED = Emergency department; FOCUS = Focused echocardiography; IVC = Inferior vena cava; LV = Left ventricle; MAP = Mean arterial pressure; POCUS = Point-of-care ultrasound; POS = Prospective observational study; qSOFA = quick Sequential organ failure assessment; SIRS = Systemic inflammatory response syndrome; US = Ultrasound; USER = Ultrasound for emergency room; VAS = Visual analog scale; vs. = Versus; yo = Years old.

**Table 3:** Case reports depicting POCUS contribution to the diagnosis and management of septic patients.

1st Author, Year, Country	Patient’ s Symptoms/Clinical Status on ED Presentation	Management and POCUS Findings	Final Diagnosis
Alhabashy, 2018, Egypt [23]	63 yo female with CAP	3 L fluids failed to improve hypoperfusion Addition of vasopressors failed to control septic shock ECHO showed AHFREF with severe aortic stenosis and mitral regurgitation	AHFREF with severe aortic stenosis and mitral regurgitation
Alonso et al., 2017, UK [24]	60-yo female, 3-day left leg pain, treated for suspected cellulitis	RUSH: severely decreased LVEF, no pericardial effusion, IVC diameter 1.2 cm with total inspiratory collapse, no free abdominal fluid MSK POCUS of left inner thigh consistent with necrotizing fasciitis	Necrotizing fasciitis
Alonso et al., 2019, UK [25]	70-yo female with diarrhea, vomiting for 1 week	Initial working diagnosis was sepsis secondary to gastroenteritis and prerenal acute kidney injury Remained hypotensive and oliguric after 2 L of IV fluids POCUS showed moderate hydronephrosis of the right kidney	Obstructive stone causing moderate right-sided hydronephrosis
Cohen et al., 2020, USA [26]	26-yo female, intravenous drug user, agitated	POCUS: hyperdynamic LV, IVC collapse > 50%, large tricuspid valve vegetation Based on labs and ECG changes, differential diagnosis included endocarditis due to *Staphylococcus*, septic pulmonary embolism, and STEMI due to embolic occlusion of the distal left anterior descending artery	Myocardial infarction caused by endocarditis-related septic embolization
Derr et al., 2012, USA [27]	69-yo male, hematemesis	POCUS: heart could not be visualized in the parasternal, apical or subxiphoid windows (suggesting pneumopericardium), free fluid and particulate matter were visualized in chest and abdomen	Esophageal perforation
Gibbons et al., 2018, USA [28]	40-yo female in severe sepsis, flank pain	POCUS: large calculus and severe hydronephrosis of the left kidney with complete loss of normal renal architecture	Xanthogranulomatous pyelonephritis
Hill et al., 2021, USA [29]	5-yo male, 2 days febrile, cough, rhinorrhea, pruritus, decreased appetite	Metabolic acidosis with hypoxemia. CXR radiopacity of the entire left hemithorax consistent with a massive pleural effusion POCUS: preserved LVEF, collapsing IVC consistent with septic shock, pleural effusion, multiple B-lines, subpleural consolidation in the right hemithorax, and complex, double-layered structure in the left hemithorax containing hypo- and hyper-echoic regions, floating in heterogeneous fluid POCUS guided proper management of septic and anaphylactic shock	Ruptured pulmonary hydatid cyst
Kinas et al., 2018, USA [30]	34-yo male, after smoking crystal methamphetamine Symptoms: palpitations, dyspnea, cough with one episode of hemoptysis	Initial resuscitation aiming at treating his tachycardia with a mixed picture of sepsis, dehydration and methamphetamine intoxication POCUS: IVC diameter 1.85 cm (maximum) in inspiration and 1.71 cm (minimum) in expiration, heart with gross biatrial and biventricular dilation, severely reduced LVEF, small pericardial effusion, lungs with bilateral lung sliding anteriorly, B-lines in bilateral inferolateral lung fields	Methamphetamine-associated cardiomyopathy
Kotlarsky et al., 2016, Israel [31]	ROS included pediatric patients with septic arthritis of the hip joint	All patients were treated with aspiration of the hip joint in the ED, with repeated aspirations as needed POCUS was used to diagnose hip joint effusion and guide aspiration, with the first one performed in the ED	Septic arthritis of the hip joint
Perez et al., 2021, USA [32]	79-yo male, with a medical history of DM, hypertension, CAD, febrile, mild dyspnea, chills, myalgias, arthralgias for the past 2 days.	MSK examination was remarkable for slight tenderness on palpation of the anterior and lateral aspect of his right shoulder MSK POCUS identified a distended, hypoechoic subdeltoid bursa, along with a communicating glenohumeral joint effusion US-guided needle aspiration of the right subdeltoid bursa was performed and the fluid analysis and culture revealed MRSA	Glenohumeral joint septic arthritis and subdeltoid septic bursitis
Romano et al., 2016, Canada [33]	61-yo female with rheumatoid arthritis, Sjogren syndrome, presented with confusion, decreased LOC, 2 weeks of productive cough, fatigue, mild dyspnea in the last 24 h	Portable CXR demonstrated an extensive heterogeneous and poorly defined right middle lobe consolidation Lung POCUS: complex loculated pleural effusion in the right posterolateral region suggestive of empyema	(Unsuspected) empyema in a patient being treated for CAP
Varela et al., 2019, Portugal [34]	77-yo male suffering from acute dyspnea, 1 week of malaise, nausea, vomiting	Fluids failed to improve blood pressure, CXR was clear POCUS: heterogeneous liver mass, pointing towards liver abscess as the cause of septic shock	Liver abscess

Abbreviations: AHFREF = Acute heart failure with reduced ejection fraction; CAD = Coronary artery disease; CAP = Community-acquired pneumonia; CXR = Chest x-ray; DM = Diabetes mellitus; ECG = Electrocardiogram; ECHO = Echocardiography; ED = Emergency department; IV = Intravenous; IVC = Inferior vena cava; LOC = Level of consciousness; LV = Left ventricle; LVEF = Left ventricular ejection fraction; MRSA = Methicillin-resistant Staphylococcus aureus; MSK = Musculoskeletal; POCUS = Point-of-care ultrasound; ROS = Retrospective observational study; RUSH = rapid ultrasound in shock; STEMI = ST-elevation myocardial infarction; US = Ultrasound; yo = Years old.

**Table 4:** Reliability indices (Sensitivity, Specificity, Positive and Negative Predictive Value) and Kappa coefficient showing degree of agreement between initial diagnosis (based on combined clinical and POCUS evaluation) and final diagnosis of distributive/septic shock.

1st Author (Year)	Shock Type	Sensitivity (%)	Specificity (%)	PPV (%)	NPV (%)	Kappa	*p*
Ahn et al. (2017) [9]	Sepsis (distributive shock)	63.6	99.7	87.5	98.7	0.729	<0.001
Bagheri-Hariri et al. (2015) [11]	Distributive	75	100	100	95.5	0.83	0.002
	Hypovolemic Distributive	100	100	100	100	1.00	0.003
Ghane et al. (2015) [12]	Distributive (RUSH Protocol)	75	100	100	94.9	0.83	0.000
Javali et al. (2020) [13]	Distributive (POCUS alone)	15	100	100	71.5	N/A	N/A
	Distributive (combined clinical and POCUS evaluation)	73.68	100	100	86.11	0.717	<0.001

## Data Availability

No new data were created or analyzed in this study. Data sharing is not applicable to this article.
